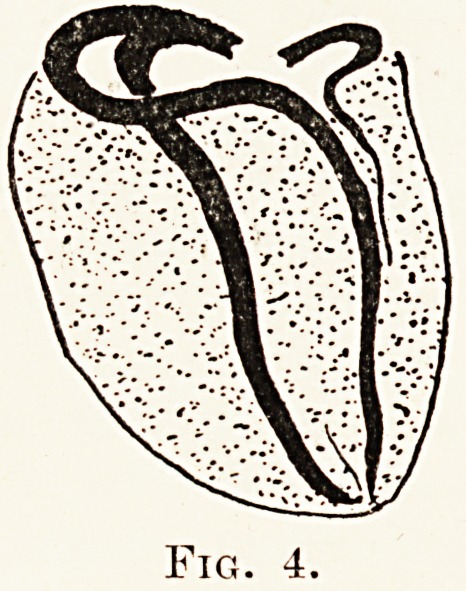# The Pathology of Coronary Occlusion
*A Paper read to the Bath and Bristol Branch of the British Medical Association, October 26th, 1927.


**Published:** 1927

**Authors:** Geoffrey Hadfield

**Affiliations:** Demonstrator of Pathology, University of Bristol; Pathologist, Bristol General Hospital


### THE PATHOLOGY OF CORONARY OCCLUSION.*

BY

GEOFFREY HADFIELD, M.D., M.R.C.P.,

*Demonstrator of Pathology, University of Bristol;*
*Pathologist, Bristol General Hospital.*

---

ALTHOUGH coronary occlusion is so frequently a cause of sudden or rapid death, it is a commonplace post-mortem observation that the heart muscle may appear normal in spite of gross sclerosis of its nutrient vessels. Even when this has produced widespread muscular cicatrisation, the age of the lesion leaves one in no doubt, not only that occlusion of a large coronary branch is often recovered from, but that the crippled heart may continue to beat for months or years after such a catastrophe—long enough sometimes for the intra-ventricular pressure to stretch the scarred walls into an aneurysmal sac.

Two fundamental problems present themselves for solution in considering the pathology of the condition; the exact cause of the sudden or rapid cardiac asystole that terminates a majority of cases, and the reason why other hearts recover from degrees of occlusion which prove fatal to the majority. In attempting to answer these questions it must first be decided to what extent the coronary arteries anastomose—a question which has been hotly debated for a hundred years.

Forty years ago Cohnheim[1] profoundly influenced

* A Paper read to the Bath and Bristol Branch of the British Medical Association, October 26th, 1927.

pathological opinion by publishing the results of his classical experiments in which the main branches of the coronary arteries were clamped in curarised dogs. The invariable result was to bring the heart to a standstill in diastole in two minutes or less. He therefore thought that no anastomoses existed between the coronary vessels, and stated that if anastomoses are present they must be of capillary calibre. The injection experiments of the time failed to show anatomical anastomoses with any degree of certainty, and for many years pathological teaching explained the asystole of coronary occlusion by assuming the production of absolute muscular ischæmia in the whole territory of the blocked vessel.

Experimental and morbid anatomical evidence then slowly accumulated which cast doubt on this assumption. It became apparent that obliteration of a coronary vessel may be followed by little or no structural change and by no obvious symptoms; that in other instances a lesion appears which is almost invariably smaller, usually much smaller, than the territory supplied by the blocked vessel; and that the blockage may produce symptoms of every degree of severity from temporary indisposition to instantaneous death.

In 1907 Spalteholtz, applying his injection and clearing technique to the study of the coronary circulation, claimed to have demonstrated that no end-arteries exist in the heart, and that there are rich anastomoses in all its muscular and endothelial layers. Cohnheim's clamping experiments were repeated, avoiding much of the trauma he inflicted, dispensing with curare and using ether, strophanthin and more careful surgical technique. The low mortality of 8·7 per cent. for ligature of the main anterior descending

### PLATE XIII.

(*a*)

(*b*)

Fig. 1.—*The coronary circulation, injected with a celloidin mass, showing free anastomoses between the two sides.*

(*a*)—From the front. (*b*)—From behind

PLATE XIV.

FIG. 2.—*Skiagram of the coronary circulation injected with bismuth-gelatin mass. Heart viewed from behind.*

branch of the left coronary artery was obtained by Miller and Mathews.[2] The mortality after tying the circumflex branch of the left coronary was reduced from 100 per cent. to 80 per cent., and to 20 per cent. for the right coronary. In all these experiments any lesions produced were appreciably smaller than the territory of the occluded vessel, except where the animal had lost much blood or where the blood pressure was lowered.

Conclusive evidence was then brought forward by Louis Gross,[3] who used a standardised injection technique, employing a radio-opaque bismuth injection mass, and either clearing the specimen or taking stereoscopic skiagrams. His published results leave one no alternative but to accept his statement that "the heart is perhaps the richest organ in the body as regards capillary and precapillary anastomoses between branches of the same main vessel and between branches from the two sides." We have obtained similar results to these by using the celloidin technique of Morison[4] followed by corrosion of the specimen in strong acid. Fig. 1 is a photograph of a preparation made by Dr. C. B. Perry in the University Cardiac Research Centre at the Bristol General Hospital. It was obtained by injecting the coronary system with X-ray film dissolved in acetone, followed by corrosion of the muscle in 25 per cent. commercial hydrochloric acid. Fig. 2 illustrates Gross's method of research, being a skiagram of a heart injected with a bismuth-gelatin mass at 300 mm. Hg. and subsequently fixed in formalin.

The existence of these anastomoses between individual branches of the coronary arteries and between the vessels of the two sides thus explains satisfactorily the recovery that may follow a large coronary occlusion in some cases. It also shows that muscular ischæmia is not the deciding factor in the production of the rapidly fatal ventricular fibrillation which so often ensues. Sudden or rapid death must therefore be due to failure of the anastomoses, rich as they are, to keep up the constant and enormous demand for blood which the heart makes on its nutrient vessels, the blood flow through which may reach 1½ litres per minute, or two or three times the volume of the heart itself (Hill).

Another factor of considerable importance is the systemic blood pressure at the time of the occlusion. As the blood pressure was lowered in experimental animals, larger and larger lesions were obtained by ligature, until a point was reached when the resulting infarct approximated closely to the territory of the ligatured vessel.

Variations in the anatomical distribution of the coronary vessels, which are often found, probably account for recovery in some cases and a fatal result in others. Fig. 3 shows the average distribution of the main vessels—the heart being viewed from behind. The main vessel of the left side is the anterior descending branch, the circumflex artery terminating as the posterior descending branch, which is relatively small. The right coronary continues as a stout vessel to the back of the heart, its anterior descending branch being relatively small and its posterior descending branch relatively large. Fig. 4 shows a common variation in which the left circumflex continues as a large vessel ending in a large left posterior descending branch, supplying a considerable part of the right ventricle, whilst the corresponding right posterior descending branch is small. An individual in whom this variation occurs has therefore a comparatively small right posterior descending branch to call upon should any of the main vessels in the left side become occluded.

One of the most interesting results of Gross's researches into the blood supply of the heart concerns the age changes which normally occur in the coronary circulation. The accumulation of epicardial fat adds to the heart a vascular sheath which slowly grows in thickness with age and makes anastomotic communications with the underlying muscle. The injection experiments we have carried out show this clearly, and there is little doubt that, provided the occlusion be slowly produced, without a serious concomitant fall in blood pressure, the anastomoses

FIG. 3.

FIG. 4.

in the epicardial fat of the senile heart help it to compensate to a considerable degree for a diminished flow through its athero-sclerotic vessels.

The lesion responsible for the narrowing or occlusion of the vessel is usually atheroma, and there is frequently a degree of calcification in the walls of the affected vessel rarely met with in visceral arteries of the same calibre in other situations. The calcification, although primarily sub-intimal, often equals in intensity the medial calcification which affects muscular arteries of the limbs. It is surprising how localised this deposit of mineral salts may be, and how frequently it affects

U

the first inch of the anterior descending branch of the left coronary and almost spares the rest of the vessel.

Although a less common cause, syphilitic arteritis accounts for a number of cases. Mesaortitis may occlude at its origin a coronary artery which is normal through the rest of its course, the orifice of the vessel in one of the sinuses of Valsalva being sometimes difficult to find in the midst of dense syphilitic scar. Should the mesaortitis be localised, this type of occlusion is easily overlooked, but is of obvious medico-legal importance.

### REFERENCES.

[1] Cohnheim and von Schulthess-Rechberg, *Virchow's Archiv*, 1881, lxxxv. 503.

[2] Miller and Mathews, *Arch. Int. Med.*, 1909, iii. 476.

[3] Louis Gross, *The Blood Supply to the Heart*, Oxford Medical Publications, 1921.

[4] Morison, *Amer. Journ. Anat.*, 1926, xxxvii. 53.

## Figures and Tables

**Fig. 1. f1:**
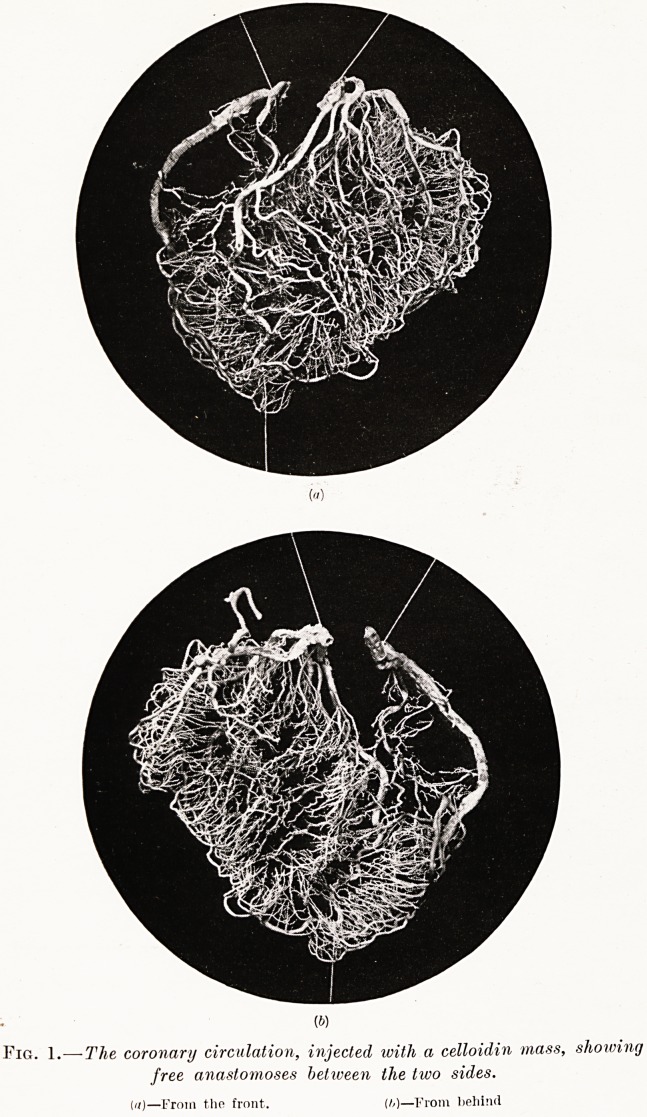


**Fig. 2. f2:**
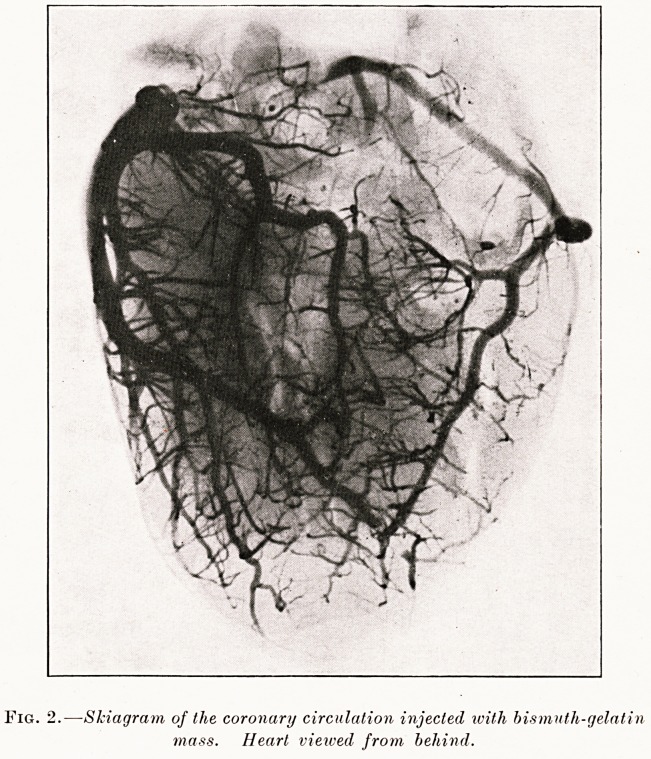


**Fig. 3. f3:**
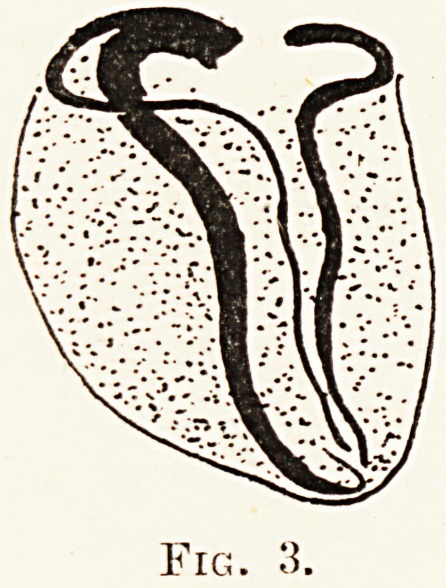


**Fig. 4. f4:**